# Antipsychotic Cardiometabolic Monitoring: Systemic Gaps and Hidden Groups

**DOI:** 10.1192/bjo.2022.317

**Published:** 2022-06-20

**Authors:** Tanya Ansari, Zafrina Majid, Ashma Mohamed, Martin Schmidt

**Affiliations:** 1 Surrey and Borders Partnership NHS Foundation Trust, Surrey, United Kingdom; 2 Head of School of Psychiatry, Surrey, United Kingdom

## Abstract

**Aims:**

To determine whether there are any gaps in cardiometabolic monitoring within primary or secondary care for people prescribed antipsychotic medication. A well-established system of cardiometabolic monitoring and checks has been implemented for patients with psychosis and bipolar in secondary care. It was unclear whether patients without these diagnoses were receiving the same level of monitoring.

**Methods:**

Data were collected retrospectively from case notes of service users under CMHRS Reigate. We included all patients from three GP practices (100 patients) and identified all who were prescribed antipsychotics and their diagnoses. The GP practices were contacted to determine whether a system was in place to flag physical health monitoring requirements for service users on antipsychotics regardless of diagnosis. The results were used to calculate the potential number of patients across the entire trust who were at risk of not receiving cardiometabolic monitoring.

**Results:**

24/100 patients were prescribed antipsychotics without a diagnosis of psychosis or bipolar. 11/24 had a diagnosis of Emotionally Unstable Personality Disorder. Quetiapine was the commonest antipsychotic. None received routine cardiometabolic monitoring.

The total caseload for all 11 adult community teams in the Trust is 2434. If prescribing and monitoring practices are similar 584 individuals may be affected.

2/3 GP practices responded. Both confirmed that they would only conduct cardiometabolic monitoring when taking over prescribing/on discharge from secondary care if specifically requested to do so.

**Conclusion:**

This service improvement project has identified a significant group of patients who aren't automatically offered cardiometabolic monitoring in secondary care.

Private correspondence from Professor David Taylor confirms that these patients would also benefit from monitoring when prescribed doses that are more likely to cause adverse effects (Quetiapine > 150mg/Olanzapine >5 mg Risperidone >2mg)

Secondary services need to identify these patients and include them in routine cardiometabolic monitoring.

Secondary services need to work closely with primary care to ensure that responsibility for checks is agreed and handed over when necessary.

